# Extracellular vesicles and soluble factors secreted by *Escherichia coli* Nissle 1917 and ECOR63 protect against enteropathogenic *E. coli*-induced intestinal epithelial barrier dysfunction

**DOI:** 10.1186/s12866-019-1534-3

**Published:** 2019-07-17

**Authors:** Carina-Shianya Alvarez, Rosa Giménez, María-Alexandra Cañas, Rodrigo Vera, Natalia Díaz-Garrido, Josefa Badia, Laura Baldomà

**Affiliations:** 1 Secció de Bioquímica i Biología Molecular, Departament de Bioquímica i Fisiologia, Facultat de Farmàcia i Ciències de l’Alimentació, Universitat de Barcelona, Barcelona, Spain; 2 Institut de Biomedicina de la Universitat de Barcelona (IBUB), Institut de Recerca Sant Joan de Déu (IRSJD), Barcelona, Spain; 3 Present Address: División Académica de Ciencias Biológicas, Universidad Juárez Autónoma de Tabasco, Tabasco, Mexico

**Keywords:** Outer membrane vesicles, *Escherichia coli* Nissle 1917, ECOR63, EPEC, Tight junctions, Intestinal epithelial barrier

## Abstract

**Background:**

Enteric pathogens have developed mechanisms to disrupt tight junctions and increase gut permeability. Many studies have analysed the ability of live probiotics to protect intestinal epithelial cells against tight junction damage caused by bacterial pathogens. *Escherichia coli* Nissle 1917 (EcN) is among the probiotics that positively modulates the intestinal epithelial barrier by regulating expression and distribution of tight junction proteins. We previously reported that regulation of ZO-1, claudin-14 and claudin-2 is mediated by EcN secreted factors, either free-released or associated with outer membrane vesicles (OMVs). Factors secreted by commensal ECOR63 elicited comparable effects in intact epithelial T-84 and Caco-2 cell monolayers.

**Results:**

Here we analyse the ability of OMVs and soluble secreted factors to protect epithelial barrier function in polarized T-84 and Caco-2 cells infected with enteropathogenic *Escherichia coli* (EPEC). Transepithelial electrical resistance, paracellular permeability, mRNA levels and subcellular distribution of tight junction proteins were monitored in the absence or presence of EcN and ECOR63 extracellular fractions. EPEC downregulated expression of ZO-1 ZO-2, occludin and claudin-14 and altered the subcellular localization of ZO-1, occludin and F-actin cytoskeleton. OMVs and soluble factors secreted by EcN and ECOR63 counteracted EPEC-altered transepithelial resistance and paracellular permeability, preserved occludin and claudin-14 mRNA levels, retained ZO-1 and occludin at tight junctions in the cell boundaries and ameliorated F-actin disorganization. Redistribution of ZO-1 was not accompanied by changes at mRNA level.

**Conclusion:**

This study provides new insights on the role of microbiota secreted factors on the modulation of intestinal tight junctions, expanding their barrier-protective effects against pathogen-induced disruption.

## Background

The intestinal epithelium constitutes a physical and biochemical barrier that limits translocation of luminal microbes, antigens, and toxins into host tissues. Several mechanisms are involved in this protective function, including production of an extracellular mucin layer, secretion of antimicrobial peptides and the formation of intercellular tight junctions that seal the epithelial layer against the luminal content, while preserving and controlling the passage of small ions and water-soluble solutes through the paracellular space. The intestinal epithelium is also crucial to orchestrate host immune responses to microbiota-derived signals [1, 2].

Adjacent epithelial cells are joined by several types of intercellular connections that include tight junctions (TJs), adherens junctions (AJs), and desmosomes. TJs are in the apical part of the polarized epithelium and regulate the paracellular permeability of the epithelial monolayer, whereas AJs and desmosomes fulfil intercellular communication functions [1]. TJs are formed by several types of transmembrane proteins such as occludin, claudins, junctional adhesion molecules and tricellulin, as well as cytosolic scaffold proteins such as zonula occludens (ZO) proteins (ZO-1, ZO-2 and ZO-3), which in turn anchor the integral membrane proteins to the actin cytoskeleton. This bridging is essential for maintaining TJ barrier integrity [3, 4]. TJs are highly dynamic structures controlled by a great variety of signaling pathways in response to multiple stimuli that regulate the expression and/or subcellular location of TJ proteins. The regulatory mechanisms include transcriptional control, membrane trafficking, and post-translational modifications that determine the ability of TJ proteins to establish specific protein-protein interactions [5–8].

Disruption of gut epithelial TJs results in enhanced intestinal permeability, a condition that positively correlates with a wide variety of diseases [9]. Enteric pathogens such as enteropathogenic *Escherichia coli* (EPEC) have evolved strategies to disrupt TJ structures as a mechanism for increasing gut permeability and helping their dissemination into the host tissues [8]. EPEC is a non-invasive pathogen whose virulence depends on a T3SS secretion system that inject virulence factors and effector proteins directly into the cytoplasm of the infected cell [10]. Translocated bacterial effectors alter functions of the target cells by different mechanisms, which involve modulation of cell signalling pathways and changes in the architecture of cellular structures such as the actin cytoskeleton, the microtubule network that directs vesicle trafficking, or connections between TJ proteins. An intricate combination of bacterial factors leads to the formation of attaching and effacing lesions on the gut mucosa, a characteristic feature of EPEC infection. Effectors such as EspF, Map and NleA contribute to TJ disruption [11, 12]. The subsequent alteration of paracellular permeability of the intestinal epithelium is one of the EPEC-mediated effects, which eventually leads to diarrhea [9, 12, 13].

Barrier disruption caused by enteric pathogens can be prevented or counteracted by certain probiotics and gut beneficial microbes [14–17]. One example is the probiotic *Escherichia coli* Nissle 1917 (EcN) with proven effectiveness in the treatment of inflammatory intestinal disorders [18–20] and acute diarrhea [21]. The EcN genome encodes a great variety of interference and fitness factors that enhance EcN survival in the gut (healthy or inflamed) and play an important role in the beneficial effects of this probiotic [22]. There is wide scientific evidence that EcN has immunomodulatory effects, mainly due to its ability to trigger activation/inactivation mechanisms of the immune response that lead to a positive balance between pro- and anti-inflammatory local cytokines [22, 23]. In addition, EcN positively modulates the intestinal epithelial barrier by triggering upregulation and redistribution of the TJ proteins ZO-1, ZO-2 and claudin-14 [24–26].

To identify the EcN factors that mediate regulation of TJs, our group was focused on the study of bacterial secreted factors, which can easily diffuse through the mucin layer before reaching intestinal epithelial cells [27, 28]. Nowadays, membrane vesicles secreted by gut bacteria are considered relevant players in microbiota-host interaction, as they allow long distance delivery of microbial effectors directly to the host [29]. We showed that outer membrane vesicles (OMVs) released by the probiotic EcN and the commensal ECOR63 are taken up by intestinal epithelial cells [30] and modulate the epithelial barrier integrity through several mechanisms. In T-84 and Caco-2 cell monolayers, vesicles from these strains reinforce TJs through activation of ZO-1 and claudin-14 expression, downregulation of the leaky protein claudin-2 and modulation of ZO-1 subcellular distribution. Free-secreted factors also contribute to this regulation [31]. In addition, EcN OMVs induce IL-22 expression in human colonic explants [32]. IL-22 acts on epithelial cells and enhances the protective function of the intestinal barrier by inducing the expression of mucin and antimicrobial proteins. In a murine model of experimental colitis, oral administration of EcN OMVs counteract the altered expression of pro-inflammatory cytokines and markers of intestinal barrier function such as Trefoil factor-3 (TFF3) [33]. This pleiotropic peptide mediates protection and repair of the epithelium by several mechanisms that include redistribution of ZO-1 to intercellular junctions [34, 35].

Based on previous studies showing that the barrier function of epithelial cell monolayers was strengthened by EcN and ECOR63 OMVs and free-secreted factors [31], here we evaluate the barrier protective effects of these extracellular fractions in a cellular model of epithelial barrier damaged by EPEC infection. The response was assessed through transepithelial electrical resistance (TER) and paracellular permeability assays, expression analysis of TJ proteins by RT-qPCR, and analysis of F-actin and TJ protein redistribution by confocal fluorescence microscopy.

## Results

### Membrane vesicles and soluble factors secreted by EcN and ECOR63 prevent EPEC-mediated epithelial barrier disruption in T-84 and Caco-2 cell monolayers

**Fig. 1:**
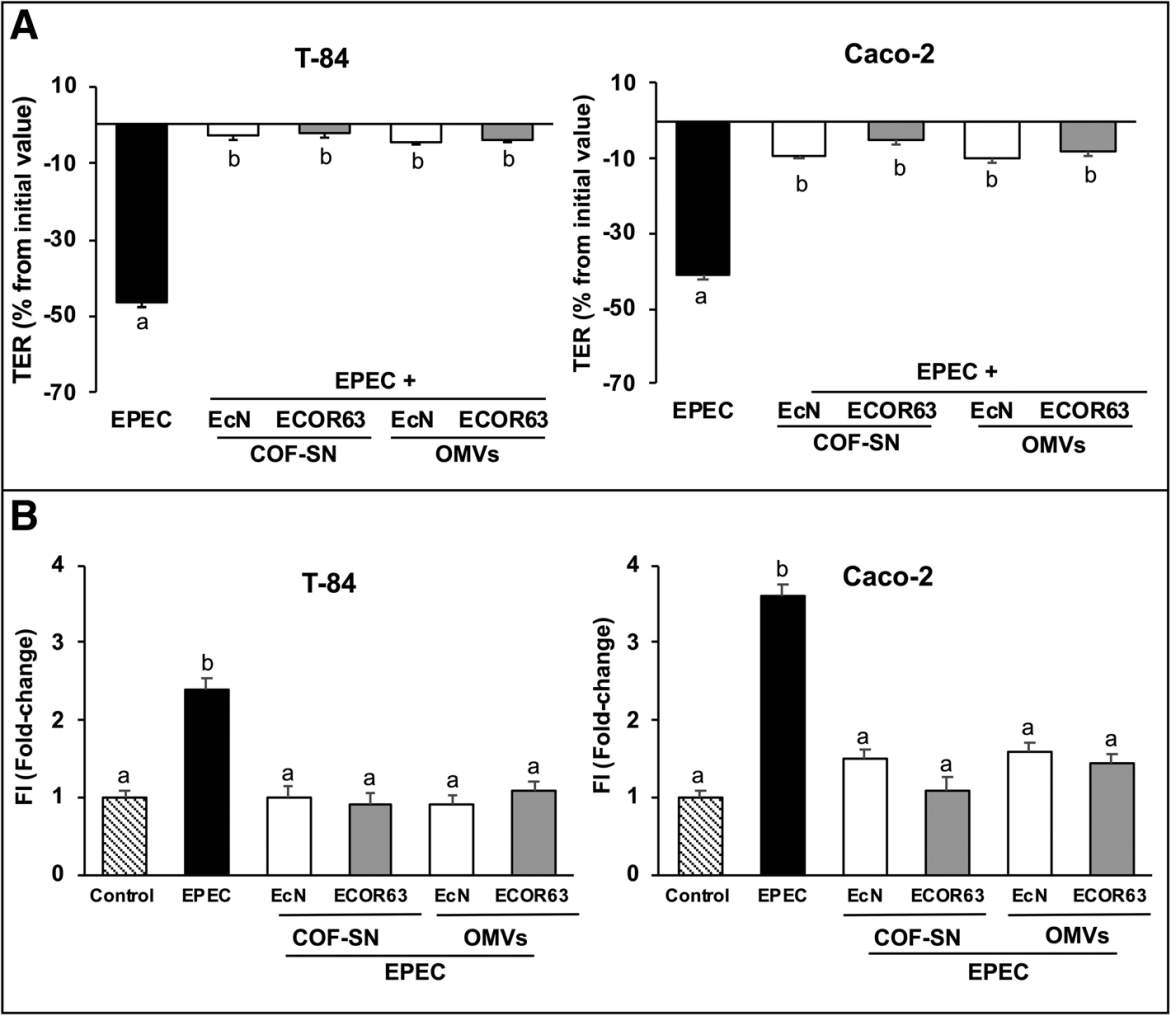
OMVs and free-soluble factors (COF-SN) secreted by EcN and ECOR63 maintain barrier function in EPEC-infected cells. Confluent T-84 and Caco-2 cell monolayers grown in Transwell supports were infected with EPEC (MOI of 100) for 3 h in the absence or presence of EcN or ECOR63 OMVs (0.1 mg/ml) or COF-SN (0.5 mg/ml). Non-treated cells were processed in parallel as a control**.** Epithelial barrier function was analysed by measuring TER (**a**) and FD-4 flux (**b**) as markers of epithelial resistance and barrier permeability. **a** TER values were measured before and after 3-h infection. Data are presented as percentage of changes in TER (decrease) from the initial value. **b** After 3-h infection, cell monolayers were washed and treated apically with FD-4 (1 mg/ml). The fluorescence in the basolateral chamber was measured before and 1 h after the addition of FD-4. FD-4 flux values were calculated by subtracting the fluorescence intensity units (FI) measured at 0 h. Data were expressed as fold-change compared with non-infected control cells, whose values were normalized as 1. In all panels, data are from three independent biological experiments performed in triplicate. The TER baseline control values were 1290 ± 98 Ω.cm^2^ for T-84 monolayers and around 920 ± 80 Ω.cm^2^ for Caco-2 cells. a, Significance against untreated control cells (*p* ≤ 0.05); b, significance against control EPEC-infected cells (*p* ≤ 0.05)

Previous studies of our group showed that cell-free supernatants collected from EcN cultures prevent disruption of Caco-2 monolayers caused by EPEC infection [36]. Therefore, the protective effect was attributed to secreted factors. Cell-free supernatants contain all the factors secreted by bacteria, either released through membrane vesicles (OMVs) or in a free-soluble form (COF-SN). As both soluble and vesicle-associated factors secreted by the probiotic EcN and the commensal ECOR63 can strengthen the epithelial barrier in cellular models that mimic the normal intestinal epithelium [31], we sought to test whether these extracellular fractions could protect against EPEC-induced damage in polarized intestinal epithelial cells. Epithelial barrier function was analysed by measuring TER and FD-4 flux as markers of epithelial resistance and permeability, respectively. To this end, Caco-2 and T-84 cell monolayers grown in Transwell membrane supports were infected with EPEC E2348/69 (MOI 100) for 3 h. In parallel, cell monolayers were infected with EPEC in the presence of OMVs (0.1 mg/ml) or COF-SN (0.5 mg/ml) obtained from DMEM cultures of EcN and ECOR63 strains. The concentration of both extracellular samples was selected according to previous dose-response curves. The same amounts were used in previous studies on intact cell monolayers [31]. Untreated cell monolayers were used as a control. EPEC infection significantly reduced the TER values of polarized cell monolayers compared to untreated control cells. However, simultaneous apical stimulation with COF-SN or OMVs from both microbiota strains neutralized the decrease in TER caused by EPEC, with TER values that did not significantly differ from those of non-infected control cells (Fig. 1a).

To examine whether OMVs and COF-SN isolated from EcN and ECOR63 also counteracted EPEC-induced increased paracellular permeability, the transport of the paracellular marker FD-4 was measured in T-84 and Caco-2 monolayers challenged by EPEC infection. In both cell lines, the unidirectional flux of FD-4 was significantly higher in cell monolayers incubated with EPEC than in controls. When cells were infected in the presence of EcN or ECOR63 extracellular fractions, there was a significant decrease in FD-4 flux with values close to those of the non-treated control cells (Fig. 1b).

**Fig. 2:**
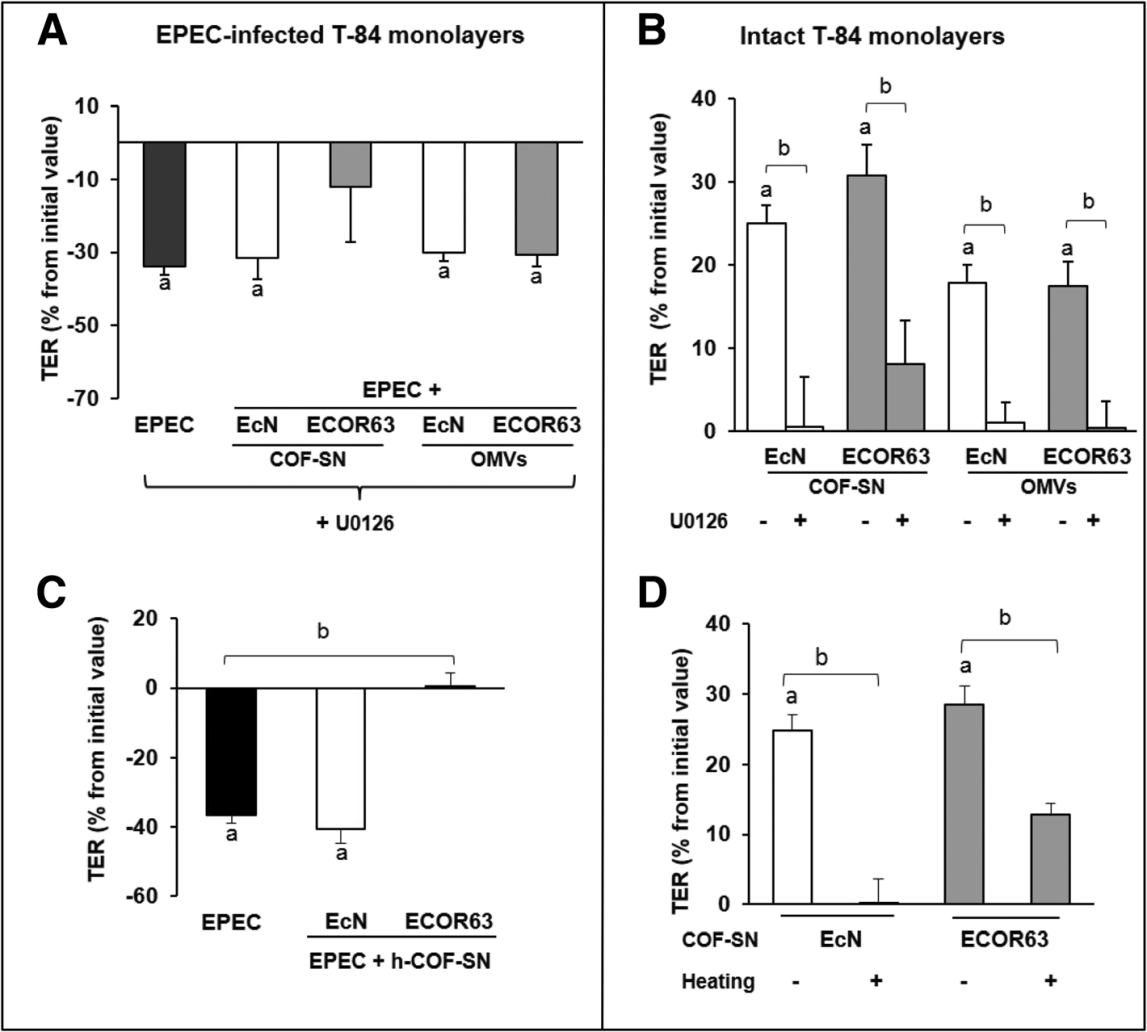
Analysis of host and bacterial factors involved in the strengthening activity of EcN and ECOR63 secreted fractions. The strengthening activity of COF-SN and OMVs depends on the ERK1/2 signalling pathway both in EPEC-infected cells (**a**) and intact cell monolayers (**b**). **a**-**b** Before infection/stimulation with OMVs or COF-SN, T-84 cell monolayers were pre-treated for 15 min with the ERK1/2 inhibitor U0126 (25 μM). TER values were measured before and after 3-h treatment. **c**-**d** Effect of heat treatment on the strengthening activity of EcN and ECOR63 COF-SN. EPEC-infected (**c**) or intact (**d**) T-84 cell monolayers were incubated with heated COF-SN (h-COF-SN), and TER values were measured before and after 3-h treatment. In all panels, data are presented as percentage of changes in TER from the initial value from three independent biological experiments performed in triplicate. TER initial values were between 1100 and 1300 Ω.cm^2^**.** a, Significance against untreated control cells (*p* ≤ 0.02); b, significance against control EPEC-infected cells (**a**, **c**) or cells treated with EcN or ECOR63 control extracellular fractions (**b**, **d**) (*p* ≤ 0.05)

The implication of the ERK pathway in the modulation of barrier integrity mediated by these bacterial extracellular fractions was evaluated in T-84 monolayers treated with the specific ERK 1/2 inhibitor U0126 (25 μM). After 15 min pre-treatment, T-84 cells were incubated with EPEC (MOI 100) alone or simultaneously with OMVs or COF-SN, and TER was measured at 3 h. In the presence of the inhibitor U0126, EcN and ECOR63 secreted fractions were unable to compensate for the decrease in TER caused by EPEC (Fig. 2a). The protective effect of all secreted fractions was virtually lost by ERK 1/2 inhibition, except for ECOR63-derived COF-SN. In this case, a tendency to neutralize the drop in TER was observed, although values did not reach statistical significance (Fig. 2a). These results indicate that the reinforcement of the epithelial barrier promoted by EcN and ECOR63 secreted factors depends, at least in part, on ERK 1/2 signalling. The regulatory effects were not restricted to conditions of damaged epithelial barrier. In fact, parallel experiments performed in T-84 cells that were not challenged by EPEC infection showed that inhibition of ERK 1/2 activity also abolishes the ability of EcN and ECOR63 extracellular fractions to increase the TER of the cell monolayers (Fig. 2b). Again, under conditions of intact epithelial barrier ERK 1/2 inhibition only resulted in partial loss of the strengthening activity of COF-SN from ECOR63.

To discover the nature of the free-soluble secreted factors that prevent EPEC-mediated reduction of TER, COF-SN were heat-inactivated before being added to the apical compartment of T-84 monolayers. As shown in Fig. 2c, heat treatment abolished the protective activity of EcN supernatants as the TER values did not differ significantly from those of single EPEC-infected cells. In contrast, the protective effect of ECOR63 COF-SN was preserved after heat inactivation. In the presence of heated ECOR63 COF-SN, EPEC-infected cells displayed TER values like those of control cells. Proteinase K treatment of EcN and ECOR63 COF-SN yielded results that were comparable with those obtained by heat inactivation (not shown). Overall, these results suggested that a protein factor mediates the protective activity of EcN COF-SN, whereas other factors of a different nature might contribute in the case of the ECOR63 supernatant. These effects were confirmed in experiments performed in non-infected T-84 monolayers incubated with heat-inactivated supernatants. Again, heat treatment completely abolished the strengthening activity of EcN COF-SN on intact cell monolayers but partially preserved the activity of the ECOR63 supernatant fraction (Fig. 2d).

### Effect of OMVs and soluble factors secreted by EcN and ECOR63 on the expression of TJ proteins in T-84 cell monolayers challenged by EPEC infection

The prevention of TER reduction in EPEC-infected epithelial cells by OMVs and COF-SN from EcN and ECOR63 pointed to counteracting changes in TJ proteins. First, we undertook RT-qPCR analyses to assess whether the protection mediated by these bacterial fractions correlated with changes in the expression of relevant TJ-proteins known to be regulated by EcN and ECOR63 secreted fractions under intact barrier conditions, such as ZO-1, claudin-14 and claudin-2 [31], or dysregulated by EPEC infection, such as occludin and claudin-1 [16].

**Fig. 3:**
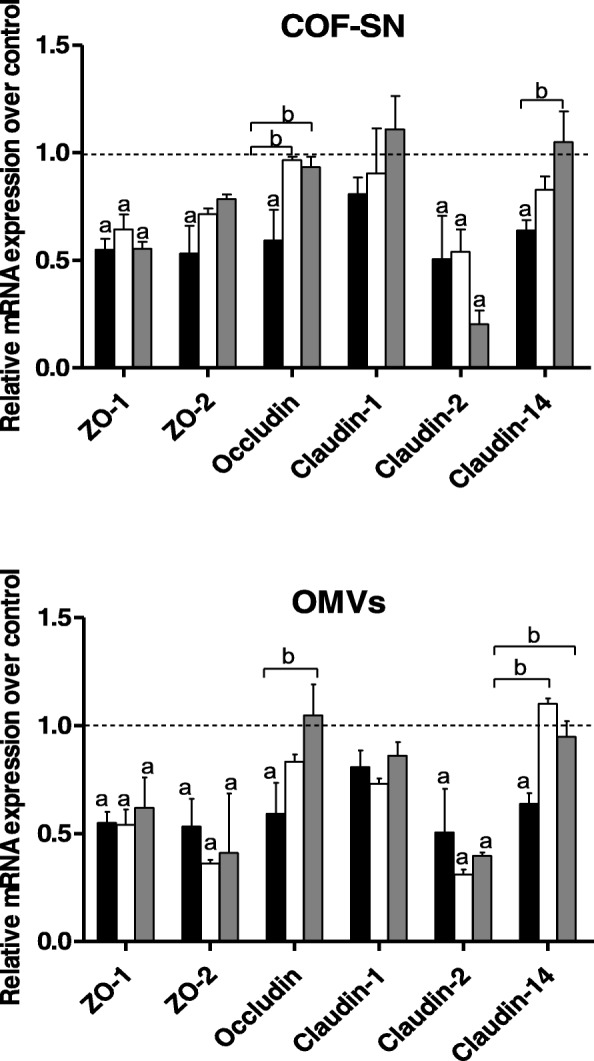
Effect of the secreted fractions (COF-SN and OMVs) from EcN and ECOR63 on the expression of TJ proteins in T-84 monolayers infected with EPEC. Intestinal epithelial cells were incubated for 3 h with EPEC at a MOI of 100 (black bars). Parallel infections were carried out in the presence of COF-SN (0.5 mg/ml) or OMVs (0.1 mg/ml) from EcN (white bars) or ECOR63 (gray bars). The relative mRNA levels of the indicated proteins were measured by RT-qPCR using β-actin as the reference gene. Data are presented as fold-change compared to untreated control cells (dotted line) from three independent biological experiments. a, Significance against untreated control cells (*p* ≤ 0.05); b, significance against EPEC-infected control cells (*p* ≤ 0.04)

For this, T-84 monolayers (9 days post-confluence) were infected with EPEC (MOI 100) for 3 h in the absence or presence of OMVs or COF-SN. Untreated T-84 monolayers were processed as a control. The relative mRNA levels of TJ proteins were measured by RT-qPCR, using the β-actin gene as a reference (Fig. 3). EPEC infection significantly decreases mRNA levels of ZO-1, ZO-2, occludin, and claudin-14, all proteins with barrier strengthening activity (Fig. 3, black bars). Interestingly, claudin-2 was also significantly downregulated by EPEC. The mRNA levels of claudin-1 were not significantly affected by EPEC infection, although a trend towards downregulation was observed compared to untreated T-84 control cells. Exposure to OMVs or COF-SN from either EcN (white bars) or ECOR63 (grey bars) during infection counteracted the EPEC-induced downregulation of occludin and claudin-14, whose expression levels remained close to those of non-infected control cells (Fig. 3). Both kinds of samples (OMVs or COF-SN), regardless of their origin (probiotic or commensal strains), had similar protective effects on occludin and claudin-14 expression, as no statistically significant differences were observed between them. In contrast, none of the microbiota secreted fractions could prevent the negative regulation of ZO-1 and ZO-2 elicited by EPEC. As expected, the low mRNA levels of claudin-2 in EPEC-infected cells remained unchanged by OMVs or COF-SN treatment. These results indicate that secreted factors released by these gut microbiota strains compensate for the EPEC-triggered negative regulation of occludin and claudin-14 expression, but not that of ZO-1 or ZO-2. Preservation of both transmembrane TJ proteins could explain, at least in part, the protection against EPEC injury observed in TER assays.

### EcN and ECOR63 secreted factors trigger redistribution of ZO-1 and occludin to tight junctions in EPEC-infected epithelial cells

After EPEC infection, occludin and ZO-1 shift from the TJ-structures located at the epithelial cell membrane to intracellular compartments. The altered distribution of these TJ-proteins greatly contributes to disruption of the intestinal epithelial barrier induced by this pathogen [12].

**Fig. 4:**
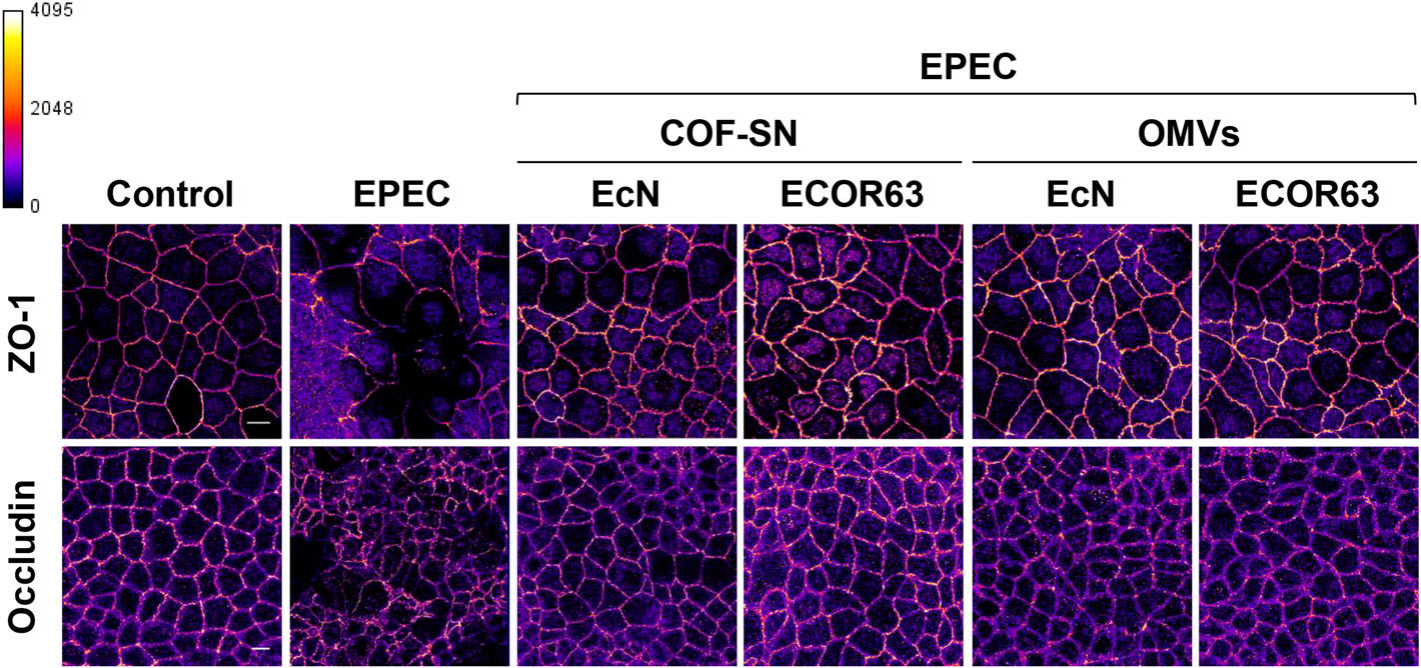
Immunofluorescence staining of ZO-1 and occludin in Caco-2 cell monolayers incubated for 3 h with EPEC in the absence or presence of COF-SN or OMVs from the indicated bacterial strains. Analysis was performed by laser scanning confocal spectral microscope with 63x oil immersion objective lens, and images were captured with a Nikon color camera (8 bit). Images shown are representative from three independent biological experiments and are coded with Fire look-up table. Calibration bar is shown on the left. Scale bar, 20 μm

To assess whether COF-SN and OMVs from EcN and ECOR63 could prevent displacement of these proteins in cells infected with EPEC we carried out an immunofluorescence microscopy analysis in Caco-2 monolayers (5–7 days after seeding). Cells were incubated with EPEC (MOI 100) for 3 h as a control for pathogen-mediated effects, and parallel infections were performed in the presence of COF-SN or OMVs from EcN and ECOR63. Then, Caco-2 cells were fixed and stained with specific antibodies against occludin and ZO-1 (Fig. 4). In cells infected with EPEC, altered distribution of both proteins was observed compared to untreated control cells.

The ZO-1 signal was mainly found in the cell boundaries of untreated control Caco-2 monolayers, whereas in EPEC-infected cells this protein was translocated from the cell membrane to the cytoplasm. The microbiota secreted fractions evaluated in this study (COF-SN and OMVs from EcN and ECOR63) showed a clear potential to neutralize the EPEC effect, thus retaining the peripheral location of ZO-1 anchored to the cell membrane.

Regarding occludin, EPEC infection triggered its dissociation from the TJ- structures. The main observation was discontinuous labelling of occludin in the cell membrane, which indicates disruption of TJs and loss of intercellular membrane contacts. In addition, a slight increase in the occludin signal in the cytoplasmic compartment was detected. When cell monolayers were infected with the pathogen in the presence of EcN or ECOR63 secreted fractions, occludin was maintained in the cell membrane with a staining profile comparable to that of untreated control cells.

**Fig. 5:**
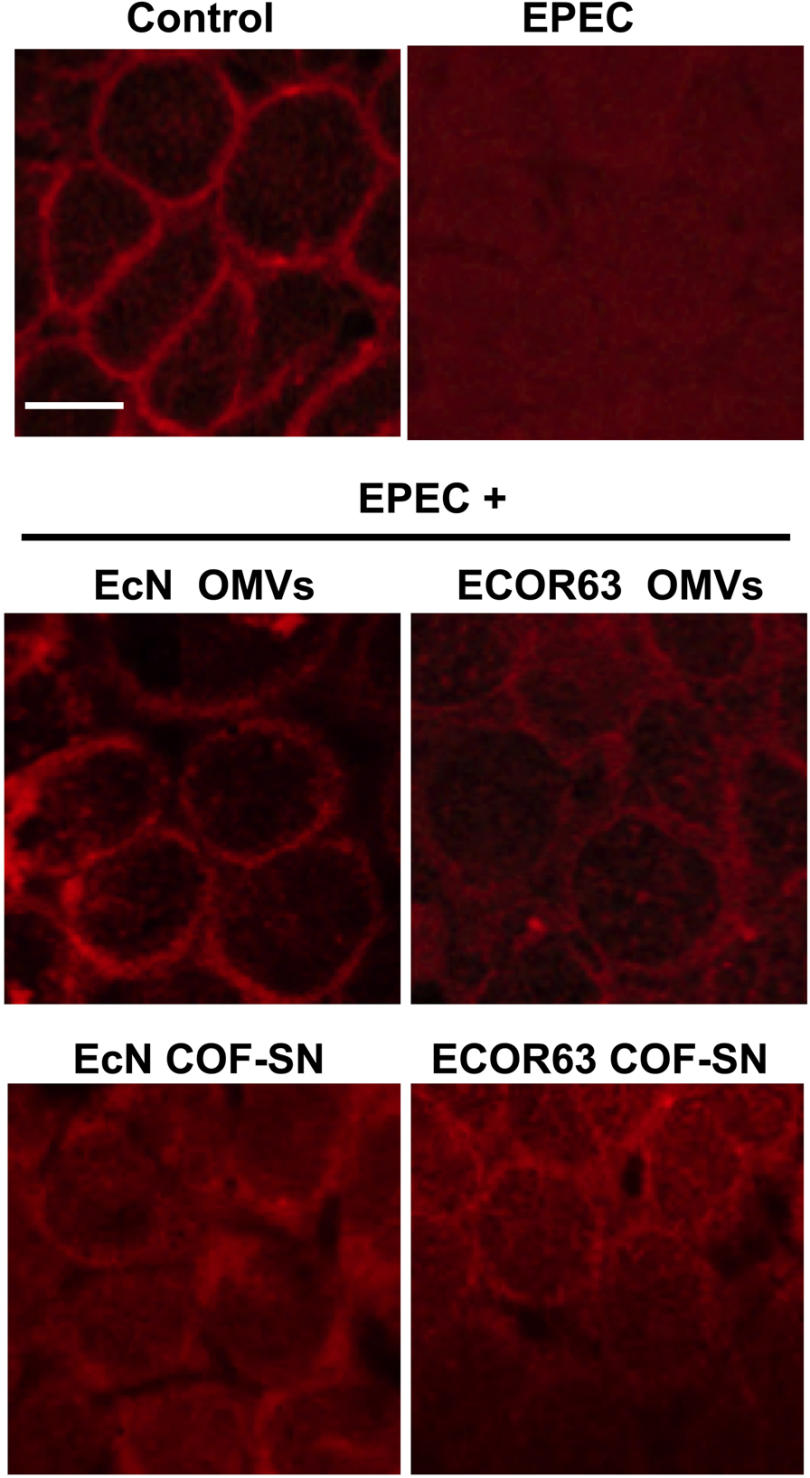
Fluorescence microscopy analysis of F-actin in Caco-2 cell monolayers incubated for 3 h with EPEC in the absence or presence of COF-SN or OMVs from the indicated bacterial strains. F-actin was stained with TRITC-labelled phalloidin (red) and analysis was performed as described for Fig. 4. Images shown are representative of three independent biological experiments. Scale bar: 10 μm

Fluorescence microscopy was also used to analyse F-actin cytoskeleton alterations. Phalloidin staining revealed a continuous distribution of F-actin at the cell boundaries of control cells. In contrast, EPEC infection caused disorganization of the F-actin architecture. The red-phalloidin fluorescence was dispersed and lost from peri-junctional areas. As for ZO-1 and occludin, incubation with OMVs or COF-SN from EcN and ECOR63 partially reversed the EPEC-induced alteration of the F-actin cytoskeleton. The compensatory effect was most relevant for the OMVs fractions. The F-actin labelling pattern clearly differed from that of EPEC-infected cells and tended to resemble the distribution pattern of the control group at the intercellular borders (Fig. 5).

Taken together, these results indicate that OMVs and free-secreted factors released by the probiotic EcN and the commensal ECOR63 can protect the intestinal epithelium against injury caused by EPEC through mechanisms that include modulation of gene expression and subcellular redistribution of TJ proteins.

## Discussion

Gut microbiota is essential to preserve intestinal homeostasis and human health. A fundamental function of this microbial community is development and maintenance of the intestinal barrier. Imbalances in microbiota composition may result in increased intestinal permeability, a condition that is linked to a wide range of illnesses [37, 38]. In the context of gastrointestinal infections, many studies have investigated the beneficial effects of certain commensal and probiotic strains in ameliorating intestinal inflammation and injury caused by several enteric pathogens [39, 40]. Enhancement of gut barrier function is among the mechanisms used by probiotics to exert their beneficial effects in the prevention and treatment of infectious diarrhoea [41]. In fact, certain probiotics protect intestinal epithelial cells against injury on TJs caused by various bacterial pathogens [14–17].

It is widely known that EPEC alters intestinal epithelial TJs, thus leading to epithelial barrier dysfunction and increased permeability [13]. Consistently, this pathogen causes reduction of TER in monolayers of several epithelial cell lines [11, 16, 25, 42, 43]. EPEC-mediated disruption of TJs has been extensively studied in several in vitro and in vivo models. These studies revealed that EPEC perturbs interaction between TJ proteins by promoting ZO-1, occludin, and claudin-1 redistribution from the intercellular junctions to a cytoplasmic location and disorganizing the peri-junctional F-actin cytoskeleton [44–46]. Several bacterial effectors injected into host cells by the T3SS act synergistically to elicit posttranslational modifications, involving phosphorylation/dephosphorylation mechanisms and the control of intracellular protein trafficking, that determine the subcellular location of the TJ proteins [12]. Moreover, EPEC also triggers downregulation of genes encoding ZO-1, occludin, and claudin-1 in the epithelial cell line NCM460 [16].

In addition to the barrier strengthening activity of EcN and ECOR63 secreted fractions (COF-SN and OMVs) on intact epithelial cell monolayers [31], the results presented here prove that these extracellular fractions also attenuate alterations in epithelial barrier function caused by EPEC infection. The ability of OMVs and COF-SN isolated from both *E. coli* strains from human gut microbiota to prevent the decrease in TER and the increase in paracellular permeability in EPEC-infected T-84 and Caco-2 cell monolayers reflected their potential to counteract lesions in TJ integrity induced by this pathogen. The ERK 1/2 signal transduction pathway is involved in the barrier protective effect of these microbial factors, as the specific inhibitor U0126 abolished their ability to reinforce intact epithelial barrier and neutralized the damage caused by EPEC in TER assays. However, some differences were observed between EcN and ECOR63, specifically in the extracellular fraction that contains free-soluble secreted factors. In the presence of the ERK inhibitor, the ECOR63 COF-SN fraction still displayed residual barrier protective/strengthening activity. This suggests that an ECOR63 free-secreted factor (not released by EcN) can modulate the epithelial barrier function through other signalling pathway(s). This observation is consistent with previous results, which showed that, in addition to free-secreted factors common to both strains, ECOR63 releases other soluble factors that have a great impact on the epithelial barrier. The common secreted factor that regulates ZO-1 and claudin-14 expression was TcpC, a protein that is not exported through extracellular vesicles protein [31]. Proteinase K and heat treatments of COF-SN fractions confirmed the protein nature of the common active secreted factor (TcpC) and the presence of other non-proteinic factors in ECOR63 supernatants. Concerning the active effectors secreted through OMVs, it should be considered that the ability of EcN and ECOR63 OMVs to reinforce the epithelial barrier is not a common feature to all *E. coli* microbiota strains, as OMVs from the commensal ECOR12 do not display such activity [31]. Bacterial vesicles enclose biological components that exist in the producer bacteria, including a high number of common microbe-associated molecular patterns, which are recognized by immune receptors in epithelial and immune cells, and also specific cargo. Therefore, some of the effects are strain-specific. In this context, proteomic studies revealed that EcN OMVs enclose specific proteins that may contribute to gut colonization and modulation of host responses [47]. Some of these vesicular proteins may mediate positive modulation on the epithelial barrier although metabolites, small RNAs or lipids cannot be ruled out. Thus, omics ‘approaches may help to identify the bioactive factors(s) secreted through OMVs.

To decipher the molecular mechanisms underlying the protective effects against EPEC infection we undertook expression and subcellular localization analysis of TJ proteins. Quantitative RT-PCR confirmed downregulation of ZO-2, ZO-1 and occludin in EPEC-infected intestinal epithelial cells [16, 25], and in addition revealed downregulation of claudin-14. However, we did not observe significant changes in claudin-1 expression. Interestingly, the mRNA levels of the leaky protein claudin-2 were diminished upon EPEC infection. Thus, unlike other enteric pathogens such as Salmonella [48], the increased permeability state induced by EPEC does not correlate with high claudin-2 levels. EcN and ECOR63 secreted fractions prevented EPEC-induced downregulation of occludin and claudin-14. In contrast, they could not counteract the EPEC-mediated decrease in ZO-1 or ZO-2 expression. Results from ZO-1 clearly differ from those reported previously in cellular models of intact epithelial barrier, which showed the ability of OMVs and COF-SN to upregulate ZO-1 expression [31]. This fact suggests that the EPEC-activated mechanisms responsible for ZO-1 downregulation could not be counteracted by these microbiota extracellular fractions. Although live EcN cells induced ZO-2 expression in T-84 cells [25], the secreted EcN fractions were unable to exert this positive effect, even under conditions of intact epithelial barrier [31]. These findings suggest that EcN-mediated upregulation of ZO-2 depends on bacteria-associated factors.

As stated above, the epithelial TJs are highly dynamic structures whose strength is regulated by multiple stimuli. Apart from transcriptional regulation [8], TJ proteins are under the control of posttranslational mechanisms that modulate their subcellular location and association with F-actin and other proteins in TJ structures. Reversible phosphorylation of TJ proteins is a crucial mechanism in the regulation of the intestinal epithelial barrier, and several kinases and phosphatases have been described to act on TJ proteins [5, 6]. The ability of EcN and ECOR63 secreted fractions to counteract delocalization of ZO-1 and occludin in EPEC-infected cells was confirmed by immunofluorescence staining followed by confocal laser scanning microscopy. For both *E.coli* microbiota strains, OMVs and COF-SN fractions protected epithelial monolayers against EPEC-induced redistribution of ZO-1 and occludin, maintaining their association with TJ structures at the cell boundaries. This suggests their potential to counteract EPEC-mediated changes in the phosphorylation state of these TJ proteins. This effect could be mediated, at least in part, by ERK activation, since occludin is a target substrate of activated ERK [6]. In Caco-2 cells, ERK-mediated phosphorylation of occludin has positive barrier effects in preventing TJ disruption by hydrogen peroxide [49]. The actin cytoskeleton is also relevant in maintaining TJ structures and modulation of paracellular solute transport in intestinal epithelial cells. Immunofluorescence analysis of F-actin revealed that EPEC-induced alterations in F-actin cytoskeleton can be partially counteracted by EcN and ECOR63 secreted fractions, specially by OMVs. As EPEC disrupts the peri-junctional actin-myosin ring by activating the myosin light chain kinase (MLCK) [50], it is likely that protection exerted by EcN and ECOR63 OMVs occurs in an MLCK-dependent fashion.

**Fig. 6:**
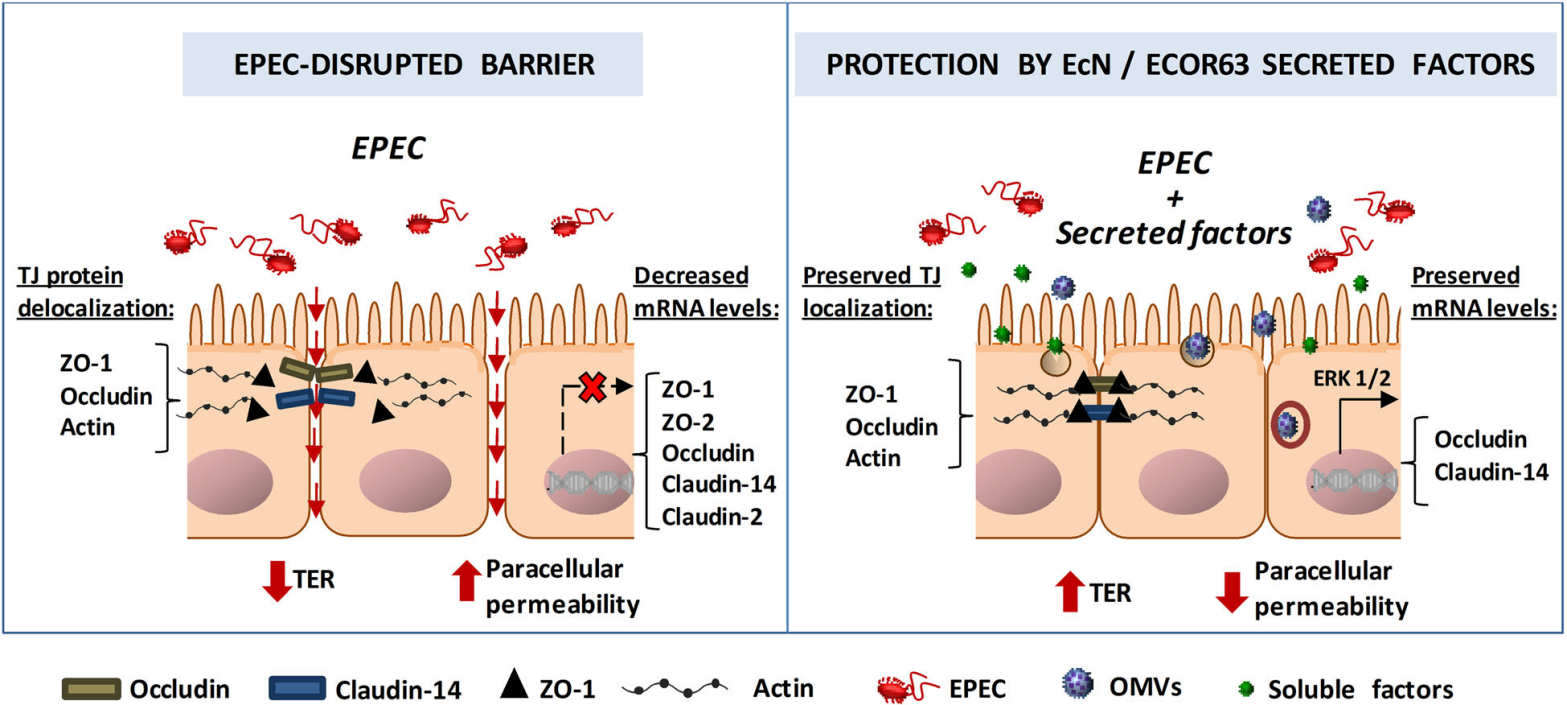
Schematic representation of mechanisms used by EPEC to disrupt the intestinal epithelial barrier and their prevention by EcN and ECOR63 secreted fractions (OMVs and soluble free-released factors)

Overall, this study reveals that the protection exerted by secreted fractions of the probiotic EcN and the commensal ECOR63 against EPEC-mediated disruption of the epithelial barrier involves compensatory regulation mechanisms at various levels (Fig. 6), targeted to: (i) mRNA expression of the TJ proteins occludin and claudin-14, (ii) redistribution of ZO-1 and occludin from the cytoplasm to TJ structures at the intercellular contacts and (iii) maintenance of the F-actin cytoskeleton structure. Considering the in vivo conditions in the human gut, it should be highlighted that crosstalk between epithelial and immune cells is crucial for maintaining intestinal homeostasis. Therefore, other mechanisms activated by EcN and ECOR63 secreted factors at the level of intestinal mucosa could synergistically protect and reinforce the epithelial barrier. In this context, the ability of EcN OMVs to upregulate IL-22 and TFF3 in colonic tissue [32, 33] may magnify the strengthening effects of the probiotic vesicles on the intestinal epithelial TJs.

## Conclusion

Microbiota-derived vesicles and secreted factors are key players in inter-species communication in the gut. Nowadays, the role of gut microbiota extracellular vesicles in health and disease is an emerging topic as they act as vehicles for the distribution and delivery of many bacterial effectors to distal tissues. Therefore, their effects are not restricted to local intestinal environment. This study broadens our current understanding of how probiotic and beneficial gut microbes exert their positive effects on the intestinal epithelial barrier. We show that OMVs and free-secreted factors by the probiotic EcN and the commensal ECOR63 can protect the barrier integrity against EPEC infection by counteracting EPEC-altered mRNA levels of occludin and claudin-14, maintaining subcellular localization of ZO-1 and occludin associated with TJ structures at the cell boundaries, and preserving F-actin at the inter-cellular junctions.

## Methods

### Bacterial strains and growth conditions

EcN (serotype O6:K5:H1) is a probiotic *E. coli* strain, obtained from Ardeypharm (GmbH, Herdecke, Germany). Strain ECOR63 was isolated from faecal samples collected from a healthy individual [51]. Wild-type EPEC strain E2348/69 (O127:H6) was kindly provided by B.B. Finlay. Cultures were grown aerobically at 37 °C in Luria–Bertani broth (LB) or in Dulbecco’s Modified Eagle Medium (DMEM) supplemented with 1% non-essential amino acids and 25 mM HEPES. Optical density at 600 nm (OD_600_) was used to monitor growth and calculate the total number of bacterial cells.

### Isolation of OMVs and cell-free supernatant fractions

OMVs and cell-free supernatants were obtained as previously described [31]. Briefly, DMEM cultures of EcN and ECOR63 were centrifuged (10,000×*g,* 4 °C, 20 min) to remove bacterial cells. The supernatants were filtered through a 0.22 μm pore size filter, concentrated with a Centricon® Plus-70 filter device and fractionated by ultracentifugation (150,000×*g,*1 h at 4 °C) into OMVs and cell-OMV-free supernatants (COF-SN). OMVs were washed and suspended in phosphate buffered saline (PBS). Samples were quantified by protein concentration [52] and sterility was confirmed by platting one aliquot on LB plates. Reproducibility of each batch of OMVs was assessed by negative stain electron microscopy [47]. When indicated COF-SN were subjected to protein-eliminating treatments. For this, aliquots of COF-SN were heated at 95 °C for 15 min or incubated with proteinase K (100 μg/ml) for 1 h at 37 °C followed by 30 min incubation with 0.5 mM phenylmethylsulfonyl fluoride.

### Cell culture and infection conditions

T-84 (CCL-248) and Caco-2 (ATCC HTB37) human intestinal epithelial cell lines were from the American Type Culture Collection. Caco-2 cells were grown in DMEM High Glucose supplemented with 10% (v/v) foetal bovine serum (FBS) and T-84 cells in DMEM/F12 Glutamax medium with 5% (v/v) FBS. In addition, culture media contained 25 mM HEPES, 1% non-essential amino acids, penicillin (100 U/ml) and streptomycin (100 μg/ml). For propagation, cells were split once a week. Then, 2 × 10^5^ Caco-2 or T-84 cells were seeded respectively in 55 cm^2^ dishes or in 75 cm^2^ flasks.

For infection, EPEC cells grown overnight in LB were diluted 1:50 with fresh medium and cultured to an OD_600_ of 0.5–1.0. Bacterial cells were collected by centrifugation and suspended in serum and antibiotic-free DMEM plus 0.5% mannose. One hour before infection, epithelial cells, grown in adequate supports depending on the experiment, were washed twice in PBS and the medium was changed to serum and antibiotic-free medium. EPEC was added to the apical side of epithelial monolayers at a multiplicity of infection (MOI) of 100 and incubated for 3 h in the absence or presence of OMVs (0.1 mg/ml) or COF-SN (0.5 mg/ml). When indicated, T-84 cell monolayers were pre-incubated for 15 min with the ERK 1/2 inhibitor U0126 at 25 μM before the addition of EPEC, OMVs or COF-SNs.

### Transepithelial resistance and paracellular marker FD-4 flux measurements

T-84 cells were seeded on the apical compartment of 12 mm polycarbonate Transwell cell culture inserts with a pore size of 0.4 μm at a cell density of 1 × 10^5^ cells/cm^2^ and cultured for 9–10 days with medium changes on alternate days. Caco-2 cells were cultured for 18–20 days following the same protocol. Assessment of monolayer integrity was followed by transepithelial electrical resistance (TER) and microscopic evaluation. TER was measured as previously reported [31]. EPEC infections were performed at initial TER values greater than 1000 Ω.cm^2^. After 3-h infection, cell monolayers were washed with PBS and kept in serum-free medium containing gentamicin 100 μg/ml for 1 h before TER measurement.

Paracellular permeability was evaluated by the flux of fluorescein isothiocyanate-labelled dextran FD-4 (4 KDa; Sigma) through differentiated cell monolayers. Prior to the experiment, monolayer integrity was checked by measuring TER. Following EPEC infection in the absence or presence of OMVs or COF-SN, cells were washed twice with PBS and fresh serum-free medium containing gentamicin 100 μg/ml was added to both Transwell compartments. Then, FD-4 was added to the apical side at a final concentration of 1 mg/ml. Aliquots of 0.1 ml were taken from the basolateral chamber at 0 and 1 h incubation and the fluorescence intensity was measured using excitation/emission wavelengths of 490/510–570 nm in a Modulus™ Microplate fluorescence reader (Turner BioSystems). For each sample, FD-4 flux values were calculated by subtracting the intensity fluorescence units measured at 0 h. Data were expressed as fold-change compared with non-treated control cells, whose values were normalized as 1.

### RNA isolation and quantitative reverse transcription PCR (RT-qPCR)

T-84 cells were grown in 12 well plates for 9 days. After 3 h infection with EPEC (MOI 100), total RNA was isolated by the TRIzol method. Concentration and purity of RNA samples were evaluated by the ratio A260/A280 measured by UV spectrometry. RNA integrity was confirmed by electrophoresis on a denaturing 1% agarose/formaldehyde gel.

For gene expression analysis, cDNA synthesis and RT-qPCR were performed as described previously [31]. Specific oligonucleotides for ZO-1, ZO-2, occludin, claudin-1 and claudin-2 are listed in Table 1. Oligonucleotides for claudin-14 were from BioRaD (PrimePCR Assay CLDN14, qHsaCED0023020). The β-actin gene was used as a reference for normalization. Appropriate control reactions were performed in parallel and melting curve analysis was applied after amplification to exclude unspecific products. All samples were amplified in triplicates to establish the mean cycle threshold value (ct). Relative changes in gene expression versus untreated control cells were calculated applying the ΔΔCt formula.

**Table 1:** Primer sequences used for RT-qPCR

Genes	Primer sequences	References
ZO-1	Fw: 5′-CGGGACTGTTGGTATTGGCTAGA-3′ Rv: 5′-GGCCAGGGCCATAGTAAGTTG-3′	[53]
ZO-2	Fw: 5′-CTAGCAGCGATCAACTTAGGGACAA-3′ Rv: 5′-CCCAGGAGTTTCATTACCAGCAA-3′	[53]
occludin	Fw: 5′- TCCTATAAATCCACGCCGGTTC − 3′ Rv: 5′- CTAAAGTTACCACCGCTGCTG − 3′	[53]
Claudin-1	Fw: 5′-GCCCCAGTGGAGGATTTACT-3′ Rv: 5′-GTTTTGGATAGGGCCTTGGT-3′	[54]
Claudin-2	Fw: 5′-ACCTGCTACCGCCACTCTGT-3′ Rv: 5′- CTCCCTGGCCTGCATTATCTC-3′	[48]
β-actin	Fw: 5′-GCTCTGGCTCCTAGCACCAT-3′ Rv: 5′-GCCACCGATCCACACAGAGT-3′	[55]

### Immunofluorescence labelling and confocal microscopy analysis

Caco-2 cells were grown in an 8-well chamber slide (Ibidi) for 6 days. After EPEC infection in the absence or presence of COF-SN or OMVs, cells were washed, fixed and permeabilized as described before [31]. Nuclei were labelled with DAPI (0.125 μg/ml), and occludin and ZO-1 were stained respectively with anti-occludin mouse IgG antibody (0.5 μg/ml, Invitrogen) and anti-ZO-1 rabbit IgG antibody (5 μg/ml, Invitrogen) [31]. F-actin was stained with phalloidin–tetramethylrhodamine B isothiocyanate (−TRITC) (dilution 1:50) as described previously [56]. Immunofluorescence was analysed in a Leica TCS SP2 confocal microscope with a 63 × 1.32NA oil immersion objective and Ar, Ar-UV and HeNe lasers. Fluorescence was recorded at 405 nm (blue; DAPI) and at 488 nm (green, Alexa Fluor 488). Images (12-bit) were obtained at a resolution of 0.232 × 0.232 × 0.488 μm/voxel (x, y, z respectively) and analysed using Fiji processing package [57].

### Statistical analysis

The SPSS software package (version 20.0, Chicago, IL, USA) was used for statistical analysis. Data were collected from at least three independent biological experiments and values are presented as the mean ± standard error (SE). The one-way ANOVA followed by Tukey’s post-test was used to assess whether differences between more than two groups were statistically significant (*p*-value ≤0.05).

## Data Availability

All data generated or analysed during the study are included in the article. Interested readers can contact the corresponding author for needed information.

## Abbreviations

COF-SN: Cell-OMV-free supernatant
DMEM: Dulbecco’s modified Eagle medium
EcN: *Escherichia coli* Nissle 1917
ERK: Extracellular signal-regulated protein kinase
FBS: Fetal bovine serum
FD-4: Fluorescein isothiocyanate-labelled 4 kDa dextran
LB: Luria-Bertani broth
MLCK: Myosin light chain kinase
OMVs: Outer membrane vesicles
PBS: Phosphate buffer saline
PCR: Polymerase chain reaction
RT-qPCR: Quantitative reverse transcription PCR
TER: Transepithelial resistance
TFF3: Trefoil factor-3
TJ: Tight junction
TRITC: Tetramethylrhodamine B isothiocyanate
ZO: Zonula occludens
